# Knowledge of trainers of retarded care centers about tooth avulsion

**Published:** 2015

**Authors:** Y Baradaran Nakhjavani, A Jafari, M Azari

**Affiliations:** * Department of Pediatric Dentistry, School of Dentistry, Tehran, Iran; ** Department of Community Oral Health, School of Dentistry, Tehran University of Medical Sciences, Tehran, Iran; *** Dentist, Tehran, Iran

**Keywords:** mentally retarded children, health educators, drop out teeth, dental trauma, educational intervention

## Abstract

Dental trauma has always been a common problem in dental health while mentally challenged children are more susceptible to it. This interventional study was conducted to evaluate the knowledge of trainers of mentally challenged children care centers about the management of dental trauma, and participants voluntarily took part in the study. A questionnaire containing demographic questions, and 12 questions related to the knowledge of trainers about the emergency management of avulsed teeth were designed. Each correct answer gave one score, and eventually, teachers who had correctly answered between 0 to 6 questions were considered to have limited real knowledge about the issue (from 7 to 9 medium, and from 10 to 12).

In the second phase, an educational intervention was done to enhance the knowledge of trainers by distributing brochures in care centers. Finally, data were analyzed by Chi-square test.

In this study, almost all participants were females (97%). Results illustrated that more experienced trainers and elder ones were relatively more knowledgeable than their younger and less experienced co-workers. Also, teachers working in private care centers had more knowledge than those working in the public sector. However, the knowledge about the management of dental traumas was unsatisfactory at first, yet it was hugely promoted after the educational intervention was carried out, which indicated the importance of conducting other educational interventions for the improvement of the management of dental traumas.

## Introduction

Dental trauma is one of the biggest problems which dental health deals with. Most dental traumas occur at the age of 2 to 3, when the child starts to walk on his own, and permanent tooth injuries are most likely to happen in 9 to 10-year-old children, as they tend to have a variety of physical activities [**1**,**2**]. The most common reasons for permanent tooth dental trauma are falling, accidents, fights, and sports involving physical activities [**2**]. One factor that makes dental trauma a real issue is parents’, health care providers’ and elementary school teachers’, and kindergarten teachers’ knowledge about how to manage dental injuries, especially avulsion [**3**-**9**]. 

An avulsed tooth may cause various discomforts such as making an inaccurate prognosis, disturbing child’s mental health, raising expenses, and complicating treatment plans. Some studies illustrate the fact that parents’ and schoolteachers’ knowledge about the ways of managing dental traumas, especially avulsion, is very limited [**4**-**6**]. On top of that, different studies shed light on the fact that knowledge about the appropriate preservation of an avulsed tooth is limited. Many developed countries have made considerable efforts to widen the general knowledge about the ways dental traumas should be managed, by designing posters, brochures, and by educational programs [**9**,**10**]. 

Children suffering from chronic epilepsy are more in danger of experiencing dental traumas. Studies prove that the significance of management of dental trauma is more highlighted when it comes to mentally challenged children, since they are more susceptible to injuries than ordinary children [**11**,**12**]. High knowledge of teachers of mentally challenged children’s care centers may have a considerable effect on preventing the dental traumas’ destructive effects. Whether an avulsed tooth is successfully replanted depends on how the periodontal ligament is conserved, which in turn, results from several factors such as including the scale of time when the avulsed tooth has been out of the socket, the media in which the tooth was kept, the material used for root cleansing, and the way the tooth was carried [**13**]. Hence, the nonprofessionals’ knowledge about the fact that the avulsed tooth needs to be correctly cleaned, and replanted within thirty minutes, or kept in an appropriate media, may hugely affect the treatment [**14**].

## Material and methods 

This was a cross-sectional and interventional study. In the first phase, all the trainers (n:137) of retarded care centers, supervised by the State Welfare Organization of Tehran Province taking care of children between 7 to 14 years old were selected. Then, a set of questionnaires was sent to the care centers’ principals, and they took the burden of distributing the questionnaires to trainers and also collecting them. The survey contained 26 questions, 14 of which were related to personal and socio-demographic information, the rest being determined to evaluate the trainers’ knowledge about the emergency of the avulsed teeth. The questionnaire was assessed by giving one score to each of the rest 12 questions that were correctly answered, if not no points were given. The knowledge was considered limited if the total score was between 0 to 6, medium from 7 to 9, and good from 10 to 12. 

An educational intervention was performed in the second phase by distributing educational brochures and pamphlets, and after three months, the knowledge of teachers was evaluated again. In this phase, a guideline for managing avulsed teeth was sent to the principals and then was given to the trainers. Teachers were asked to read the instructions carefully. The guidance contained information about: 1) Problems and side effects of dental trauma. 2) Factors that escalate the risk of going through a dental trauma, 3) Emergency measures that everyone can take. 4) The role of time in enhancing treatment prognosis. 5) Necessary actions in dental trauma emergency on both hard and soft tissues. 6) Identifying children with high risk of facing a dental trauma. After three months, questionnaires were sent to the principals by the State Welfare Organization of Tehran Province and trainers were asked to answer the questionnaire in the presence of the head. Filled questionnaires were sent back to the Organization and subsequently scored. Results were compared to the product of the first set of polls to see if they have improved. Chi-square test was used to evaluate the effect of each variable in enhancing or decreasing the knowledge score of teachers. 

## Result

One hundred and thirty-seven trainers who worked in care centers for mentally challenged children, who were under the supervision of State Welfare Organization of Tehran Province, participated in this quest. Only five teachers were males, and the rest were females. Results illustrated that more than one-fifth of trainers, who were all females, had good knowledge about managing an avulsed tooth. Both elder and more experienced teachers had more experience than younger and less experienced ones (P < 0.001). 

Thus, it can be stated that there was a significant relation between the teachers’ academic background and their knowledge, since trainers that were more educated did not have more knowledge in this area (P > 0.05). Furthermore, the type of the care center had a significant role in varying the scale of the trainers’ knowledge in a way that teachers working in a private care center had more knowledge regarding the issue than those working in a public one. Moreover, almost all the teachers (95%) supervised children while playing and this did not have any relationship with their level of knowledge. Also, trainers who attained a First Aid certificate were significantly more knowledgeable than those who did not (**Table 1**). Furthermore, it was seen that almost all the trainers (96%) observed kids while playing, and more than 1 out of 10 persons (13.8%) had enough knowledge about the appropriate type of media, which had to be used for the preservation of the avulsed tooth.

**Table 1:** Level of knowledge and variables among trainers of retarded care centers in Tehran about tooth avulsion

Level of Knowledge\Variable		Good (%)	Average (%)	Weak (%)
Sex	Male	0	0	0
	Female	30	44	58
Age	20-30	17	13	29
	31-40	8	16	16
	>40	5	15	18
Level of Education	Below high school diploma	9	15	14
	High school diploma	9	18	31
	Academic	12	11	18
Work Experience (years)	<5	15	16	35
	5-10	9	10	13
	>10	6	18	15
Type of the working area	Public	17	31	24
	Private	13	13	39
First Aid Certificate	Yes	19	27	27
	No	11	17	36
Experience of facing an avulsion	Yes	1	8	0
	No	29	36	63

Brushing the previous facts away, results illustrated the fact that an educational intervention can affect the teachers’ knowledge score. After the intervention was carried out in the second phase, the results altered in the following way: 61% of the teachers had the right experience, 33% had the adequate knowledge, and 6% still had limited knowledge regarding the issue. 

## Discussion

Notwithstanding the fact that nowadays almost all lost dental tissues can be repaired, losing a permanent tooth is a traumatic experience, since the treatments are usually too complicated and expensive. Despite the precautions taken by parents and teachers, dental traumas are still inevitable, and, in most cases, the emergency is in the hands of an ignorant person, and these individuals manage the crisis until professionals take care of the problem. Hence, it is required to be aware of the essential role of ignorant people in managing dental traumas. This study was performed to evaluate the scale of trainers’ knowledge working in care centers for mentally challenged children in Tehran, Iran, about the management of the emergency of avulsed teeth, along with an educational intervention. 

One hundred and thirty-seven trainers working in care centers with children between 7 to 14 years old took part in this study. Almost half of the trainers had limited knowledge about the management of the avulsed teeth, while only 22% had a good understanding in this area. 

Results illustrated that there was no relationship between the intelligence score of trainers, and observing the kids while playing, and only 4% of the teachers had no observation on children while frolicking. Other studies considered this fact as well. A study in the United Kingdom showed the same results, while more than 97% of the teachers kept observing children play under surveillance [**7**]. In this study, more than half of the trainers who watched students did not have adequate knowledge about the management of dental trauma in the first phase; however, the total score knowledge was increased after the intervention. Besides, other studies revealed the fact that many parents and school teachers also had a paucity of understanding in the field of dental trauma management [**4**,**6**]. Moreover, another study in Hong Kong reported that the age of the teachers had no significant effect on their knowledge since they all had enough experience in this realm [**10**]. In our study, the location of the care center significantly affected the knowledge of trainers. Also in a Singapore study, since the majority of teachers had an academic education, the relationship between the level of teaching and the scale of knowledge was not significant, although, in this study, the standard of education had a significant effect on the extent of knowledge. This study showed that knowledge and working experience have a direct relationship, likewise other studies in Singapore and England [**3**,**15**]. 

Another known truth about the emergency management of avulsion is that there is a shortage of knowledge about preserving the avulsed tooth in an appropriate media. While in this study 13.8% of the trainers would choose the best media to keep the tooth, a research conducted in Turkey proved that less than 10% of the parents have adequate knowledge about the press in which the avulsed tooth should be held, and also the way an avulsed tooth should be cleansed [**5**]. Furthermore, in India, Namdev et al. stated in their study, that among the total 1500 participants, 566 parents would rather keep an avulsed tooth in a piece of paper tissue or handkerchief, and one-third of them would rather keep the tooth in a plastic wrap [**16**]. Another Indian study showed that 80% of the parents did not use any media to preserve the avulsed tooth [**17**]. For instance, a study in Hong Kong showed that primary and secondary school teachers have limited knowledge about dental trauma, especially about avulsed tooth management, and about the adequate media in which the avulsed tooth should be kept [**18**]. Besides, another survey in Belgaum, India, reported that only 2.2% of the nurses working in KLE Institute of Nursing Science knew about the right media in which an avulsed tooth needed to be preserved. Most nurses admitted that they did not have adequate knowledge about avulsion management and that they had no previous training in this field [**19**]. 

Moreover, in this study, more than half of the trainers (53%) attained a first aid course, which had a positive effect on their knowledge towards the management of dental traumas, although other studies had a much better record in this field. Studies in Hong Kong and England showed that 99% and 91% of the teachers had passed a first aid course respectively [**10**,**15**]. Although a study in Jordan showed that even though almost half of the teachers had attained a first aid course once during their teaching career, only 5% were trained in dental first aid. Hence, taking a first aid course does not necessarily mean being familiar with the principals of dental first aid [**20**].

The incidence of dental traumas in Singapore, Hong Kong, and England was 24%, 28.3%, and 34.7% respectively [**3**,**10**,**15**]. Also, a study in India amongst Maharashtrian Population showed that 90% of the children had suffered from dental trauma [**17**]. Proceeds of the studies mentioned above revealed the fact that dealing with the emergency of an avulsed tooth has no significant effect on knowledge regarding the management of dental traumas. Thus, it can be concluded that if the scale of knowledge was high, confronting dental injuries may have no significant effect on the experience. 

Many studies emphasized on educational interventions should be conducted to improve the level of knowledge about dental trauma management. The Jordanian study stated that most mothers are keen on receiving further edification in this field [**21**]. 

Studies revealed the fact that mentally challenged children are more susceptible to dental traumas than ordinary children due to their movement problems [**11**,**12**]. As mentioned earlier, mentally challenged children are more vulnerable to dental injuries, and are also less able to express themselves; hence, are more in need of receiving attention from dental authorities. Educational interventions have always hugely affected the dental health of societies. A survey of secondary students in Hong Kong showed that posters play an imperative role in enhancing students’ knowledge about the emergency management of dental trauma. Based on the results of this study, the awareness of the intervention group who had access to posters containing information about dental trauma management in their classroom was significantly improved during a two-week intervention [**18**]. In our study, the knowledge of trainers ascended dramatically after the educational intervention, with 61% having sound knowledge about the issue, versus 22% before the intervention was performed, confirming that the program was a success. Besides, it showed the importance of educating teachers and trainers in elementary schools and kindergartens in Iran based on the high incidence of dental traumas.
